# Successive clinical application of vitamin D and bumetanide in children with autism spectrum disorder: A case report

**DOI:** 10.1097/MD.0000000000018661

**Published:** 2020-01-10

**Authors:** Jun-Yan Feng, Hong-Hua Li, Bing Wang, Ling Shan, Fei-Yong Jia

**Affiliations:** a Department of Developmental and Behavior Pediatrics, The First Hospital of Jilin University; b Neurological Research Center of First Hospital of Jilin University, Changchun, China.

**Keywords:** autism spectrum disorder, bumetanide, vitamin D

## Abstract

**Rationale:**

Autism spectrum disorder (ASD) is a common neurodevelopmental disorder caused by complex interactions between genetic and environmental factors. Recent studies suggest that Vitamin D_3_ or bumetanide therapy may improve the core symptoms of ASD in some individuals. However, there are no guidelines that provide clinicians with evidence-based treatment regimens for the use of these therapies in ASD.

**Patient concerns:**

A 30-month-old female was referred to our department because she did not respond when her name was called.

**Diagnosis:**

The patient was diagnosed with ASD by a team of autism experts according to American Psychiatric Association Diagnostic and Statistical Manual of Mental Disorders (DSM-5) criteria.

**Interventions:**

The patient was administered Vitamin D_3_ 150,000 IU intramuscularly once a month and Vitamin D_3_ 800 IU orally each day. After 6 months, Vitamin D_3_ supplementation was discontinued because of lack of effectiveness. Subsequently, oral bumetanide 0.5 mg twice daily was initiated.

**Outcomes:**

The patient's symptoms remained unchanged after 6 months of Vitamin D_3_ supplementation, and her serum 25 (OH) D levels had reached 52.4 ng/mL. At the parent's request, Vitamin D_3_ supplementation was discontinued because of lack of effectiveness. Thereafter, bumetanide was initiated. After 1 month of bumetanide, the patient's Childhood Autism Rating Scale score was 26, which is below the cutoff score for ASD. This case report suggests that Vitamin D_3_ and bumetanide target different mechanisms in the pathogenesis of ASD.

**Lessons:**

Based on these observations, we discuss three possible scenarios for Vitamin D_3_ supplementation and propose that bumetanide should be initiated if Vitamin D_3_ supplementation is ineffective (identifier ChiCTR-CCC-13004498).

## 1 Introduction

Autism spectrum disorder (ASD) is defined as a group of neurodevelopmental dysfunctions that affect social interaction and communication and result in restricted interest and stereotyped repetitive behaviors. The prevalence of ASD has steadily increased from 1/2500 in 1984 to 1/68 in 2014.^[1]^

Management of ASD includes education, supportive care, and behavioral therapy. Antipsychotics, stimulants, and antidepressants are prescribed adjunct to behavioral therapy to treat related conditions and problem behaviors in ASD, including depression, anxiety, hyperactivity, and obsessive-compulsive behaviors. Currently, there is no cure for ASD.

The pathogenesis of ASD results from complex interactions between genetic (mutation, copy number variation, chromosomal abnormality) and environmental (folic acid deficiency, antiepileptic drugs, food allergy) factors.^[1]^ Previous studies have identified Vitamin D deficiency as a risk factor for ASD^[2]^ and indicate that Vitamin D supplementation can ameliorate the core symptoms of ASD in children,^[3,4]^ providing a novel approach to therapeutic management of this patient population.

γ-aminobutyric acid (GABA) is a neurotransmitter that shows an excitatory/inhibitory shift during brain maturation.^[5]^ This shift is mediated by the developmentally regulated expression of the chloride importer NKCC1 and the chloride exporter KCC. There is a deficit of inhibitory GABA in patients with ASD. Benzodiazepine, a GABA receptor agonist, produces paradoxical effects in children with autism, suggesting that the maturation of GABAergic neurons in the brain of these patients is delayed or insufficient. Bumetanide, a NKCC1 antagonist, can reduce intracellular chloride, shift GABAergic responses from excitation to inhibition, and improve the clinical symptoms of ASD in children.^[6]^

Both Vitamin D_3_ and bumetanide are relatively safe, readily available, and inexpensive, making them attractive for use in clinical practice. However, there are no guidelines that provide clinicians with evidence-based treatment regimens for the use of these therapies in ASD. To inform clinical practice, we report our clinical experience of Vitamin D_3_ and bumetanide therapy in the case of a child with ASD.

## 2 Case presentation

A 30-month-old female was referred to the Department of Developmental and Behavioral Pediatrics at our institution (Changchun, China) on December 2, 2017 because she did not respond when her name was called. The patient was diagnosed with ASD by a team of autism experts according to Diagnostic and Statistical Manual of Mental Disorders (DSM-5) criteria developed by the American Psychiatric Association. Her symptoms included

1) Social and communication difficulties: no urge for social interaction, no response when somebody called her name, no social smiling, not following instructions from her parents, avoidance of eye contact and physical contact, inability to properly play with and share with peers.
2) Qualitative deficits in language development and communication: lack of verbal communication.
3) Restricted/stereotypic behavior and restricted interests: biting objects like an infant, inability to properly play with toys, hyperactivity manifest as endless running and circling.

The child also suffered from insensitivity to painful stimuli such as falling down a staircase, and had urinary and fecal incontinence.

There was no family history of psychiatric or central nervous system disorder. A brain magnetic resonance image and 24-hour ambulatory electroencephalogram monitoring were normal. Karyotype analysis was normal (46 XX). Clinical chemistry analyses of serum and urine were normal. The patient's Childhood Autism Rating Scale (CARS) score was 31. The CARS is a 7-item scale that is used to assess the severity of autism, where 30 is the cutoff score for autism, 30 to 33 indicates mild to moderate autism, and > 37 indicates severe autism. Serum 25-hydroxyvitamin D [25(OH)D] level on high-performance liquid chromatography was 19.4 ng/mL. Serum 25(OH)D levels ≥ 30 ng/mL are considered adequate, < 30 and > 10 ng/mL are considered inadequate, and ≤10 ng/mL are considered deficient.^[4]^

The patient was administered Vitamin D_3_ 150,000 IU intramuscularly once a month and Vitamin D_3_ 800 IU orally each day. At the first follow-up visit (February 8, 2018), 2 months after treatment initiation, the patient's parents reported no change in her symptoms of autism, but her serum 25(OH)D level had increased to 38.1 ng/mL. Vitamin D administration was continued until the second follow-up visit on June 12, 2018. The patient's mother confirmed there had been little improvement in the child's symptoms of autism, her CARS score remained at 31, but the patient's serum 25(OH)D level had increased to 52.4 ng/mL. At the parent's request, Vitamin D_3_ supplementation was discontinued because of lack of effectiveness. Subsequently, oral bumetanide 0.5 mg twice daily was initiated. Prior routine laboratory blood and urine tests confirmed liver function and hematology were unremarkable. During the follow-up visit one month later (July 15, 2018), the patient's mother reported that her daughter's symptoms were improved. After only 1-week of bumetanide, the patient showed positive language development such as starting to speak voluntarily (e.g., “mama”). Her hyperactivity was reduced, and she was more responsive when someone called her name. After 1 month of bumetanide, the patient's behavior is significantly improved except for sometimes avoiding eye contact and insensitivity to painful stimuli. The patient was followed up on April 29, 2019. She has been attending kindergarten, can listen to her teacher's instructions, is motivated to actively communicate with other children, and can express herself with appropriate language. Occasionally, the patient uses imitation and stereotyped language when she does not understand instructions, and she has heightened sensitivity to eye contact and pain stimuli compared to before bumetanide. The patient shows no restricted and repetitive interests and activities. Her latest CARS score was 20. There were no clinical abnormalities in the patient's blood or urine following bumetanide treatment. Further follow-up is ongoing.

## 3 Discussion

In this case, we report our clinical experience of Vitamin D_3_ and bumetanide therapy in a child with ASD. To our knowledge, this is the first case study showing that bumetanide administration markedly improved symptoms of ASD after vitamin D supplementation proved to be ineffective. Vitamin D is a fat-soluble vitamin that is present in the diet in limited amounts. Vitamin D is obtained mainly by skin exposure to UVB radiation. A growing body of evidence suggests that Vitamin D deficiency is more prevalent in children with autism compared to healthy controls.^[2]^ Several studies have shown that Vitamin D plays an important role in brain development and function. Therefore, Vitamin D deficiency may be involved in the pathogenesis of neurodevelopment disorders such as intellectual disability, developmental delay, multiple complex disability disorder, and ASD.^[7]^ Mechanistically, Vitamin D may contribute to the abnormalities associated with the etiology of ASD, such as de novo gene mutations, oxidative stress, impaired detoxification, inflammation, immune modulation, and abnormal neurotrophic factor and neurotransmitter levels.^[8]^

Based on previous data, Cannell et al proposed a possible relationship between Vitamin D deficiency and autism and predicted that autism symptoms might be ameliorated with Vitamin D supplementation. Our published reports ^[3,4]^ have confirmed this hypothesis. Vitamin D may improve the clinical symptoms of ASD by acting as an antioxidant, immune modulator, and regulator of gene expression and serotonin synthesis in the brain.^[9]^ In this study, the patient had a low serum 25 (OH) Vitamin D level when she was referred to our clinic. Vitamin D supplementation was chosen as the initial treatment option for this patient as Vitamin D is easily available, inexpensive, and relatively safe. The clinical symptoms of our patient remained unchanged after 6-months of Vitamin D supplementation, even though the patient's serum 25(OH)D concentration had reached 52.4 ng/mL.

However, bumetanide administration markedly improved ASD symptoms in this patient. Bumetanide is a loop diuretic that is usually administered as an anti-hypertensive as it inhibits sodium transport in the thick ascending limb of the loop of Henle.^[10]^ In neurons, bumetanide antagonizes the chloride importer NKCC1 to reduce internal chloride concentrations, terminating GABA excitation and initiating GABA inhibition. Recent studies have shown that bumetanide is effective for improving the clinical symptoms of ASD.^[5,6]^ Animal experiments demonstrated that bumetanide exerted its effects by inhibiting the GABAergic signaling pathway.^[11]^ Taking this into consideration, and complying with the request of the patient's parents to discontinue Vitamin D supplementation, bumetanide therapy was initiated. At just 1 week after bumetanide administration, the patient began to speak voluntarily and sometimes said “mama”. She also responded if someone called her name. The patient's hyperactive symptoms were improved within one month and her latest CARS score dropped to 20, which is below the cutoff score for ASD.

Findings from our previous reports suggest that supplementation with Vitamin D_3_ is a safe and cost-effective treatment that may significantly improve the outcome of some children with ASD, especially younger children.^[3,4]^ Hadjikhani et al showed that bumetanide treatment improves emotion recognition and enhances the activation of brain regions involved in social and emotional perception during the perception of emotional faces, reinforcing the usefulness of bumetanide as a promising treatment to improve social interactions in autism.^[6]^

In the present case, bumetanide administration markedly improved symptoms of ASD after Vitamin D_3_ supplementation proved to be ineffective, implying that Vitamin D and bumetanide target different mechanisms in the pathophysiology of ASD. Therefore, we believe that ASD can be divided into different subtypes. Among these, one subtype may respond to treatment with Vitamin D_3_ and one subtype may respond to bumetanide. The patient in this report may have deficits in the GABAergic system and respond to bumetanide. Further studies are needed to fully elucidate the pathophysiology of ASD.

Currently there are no guidelines for administering Vitamin D_3_ or bumetanide therapy in children with ASD. Consequently, we propose the following 3 scenarios:

1. Vitamin D_3_ supplementation should be initiated in children with ASD and Vitamin D insufficiency, with regular monitoring of Vitamin D status. This scenario assumes that children with ASD and normal serum 25(OH)D levels do not benefit from Vitamin D_3_ supplementation. However, our study suggests that ASD symptoms are present even when serum 25(OH)D levels are high.
2. Vitamin D_3_ supplementation should be initiated in children with ASD and Vitamin D deficiency, with regular monitoring of Vitamin D status. This scenario assumes that ASD symptoms are only associated with Vitamin D deficiency.
3. Vitamin D_3_ supplementation should be continued for at least 6 months. The risk for over-dosage is negligible, and only occurs when Vitamin D_3_ is supplemented at 100-fold above the recommended dosage. Considering the availability of Vitamin D_3_ and that it is a safe and cost-effective treatment, Vitamin D_3_ supplementation could be recommended as a first-line treatment choice for children with ASD. If this fails, bumetanide would be the alternative.

In conclusion, this case report demonstrated that Vitamin D supplementation and bumetanide therapy might influence the core symptoms of ASD through different mechanisms. Vitamin D supplementation could be considered as a first-line treatment choice for children with ASD and inadequate serum Vitamin D level. If this fails, bumetanide represents a reasonable second-line intervention. However, in the absence of rigorous randomized controlled studies and follow-up studies, which are urgently needed to generate evidence-based clinical recommendations for the management of ASD symptoms, Vitamin D and bumetanide should currently be considered disease-modifying measures for ASD.

## Acknowledgments

We would like to thank Medjaden Bioscience Limited for editing and proofreading this article.

## Author contributions

**Conceptualization:** Jun-Yan Feng, Fei-Yong Jia.

**Data curation:** Jun-Yan Feng, Hong-Hua Li, Bing Wang, Ling Shan.

**Investigation:** Jun-Yan Feng, Hong-Hua Li, Ling Shan, Fei-Yong Jia.

**Project administration:** Fei-Yong Jia.

**Resources:** Bing Wang.

**Supervision:** Jun-Yan Feng, Fei-Yong Jia.

**Validation:** Hong-Hua Li, Bing Wang, Ling Shan, Fei-Yong Jia.

**Visualization:** Jun-Yan Feng, Hong-Hua Li, Bing Wang, Ling Shan, Fei-Yong Jia.

**Writing – original draft:** Jun-Yan Feng.

**Writing – review & editing:** Jun-Yan Feng, Hong-Hua Li, Bing Wang, Ling Shan, Fei-Yong Jia.
